# Tracking from the Tropics Reveals Behaviour of Juvenile Songbirds on Their First Spring Migration

**DOI:** 10.1371/journal.pone.0105605

**Published:** 2014-08-20

**Authors:** Emily A. McKinnon, Kevin C. Fraser, Calandra Q. Stanley, Bridget J. M. Stutchbury

**Affiliations:** Dept. of Biology, York University, Toronto, Ontario, Canada; Pennsylvania State University, United States of America

## Abstract

Juvenile songbirds on spring migration travel from tropical wintering sites to temperate breeding destinations thousands of kilometres away with no prior experience to guide them. We provide a first glimpse at the migration timing, routes, and stopover behaviour of juvenile wood thrushes (*Hylocichla mustelina*) on their inaugural spring migration by using miniaturized archival geolocators to track them from Central America to the U.S. and Canada. We found significant differences between the timing of juvenile migration and that of more experienced adults: juveniles not only departed later from tropical wintering sites relative to adults, they also became progressively later as they moved northward. The increasing delay was driven by more frequent short stops by juveniles along their migration route, particularly in the U.S. as they got closer to breeding sites. Surprisingly, juveniles were just as likely as adults to cross the Gulf of Mexico, an open-water crossing of 800–1000 km, and migration route at the Gulf was not significantly different for juveniles relative to adults. To determine if the later departure of juveniles was related to poor body condition in winter relative to adults, we examined percent lean body mass, fat scores, and pectoral muscle scores of juvenile versus adult birds at a wintering site in Belize. We found no age-related differences in body condition. Later migration timing of juveniles relative to adults could be an adaptive strategy (as opposed to condition-dependent) to avoid the high costs of fast migration and competition for breeding territories with experienced and larger adults. We did find significant differences in wing size between adults and juveniles, which could contribute to lower flight efficiency of juveniles and thus slower overall migration speed. We provide the first step toward understanding the “black box” of juvenile songbird migration by documenting their migration timing and en route performance.

## Introduction

Optimal migration timing and stopover habitat selection are critical for migratory animals, since mistiming their migration relative to peaks in resource abundance at breeding sites can have negative fitness consequences [1]–[4]. Mismatched migration timing, e.g. making landfall in resource-poor stopover habitat, can also affect survival during migration itself [5], or have non-lethal carry-over effects that influence subsequent breeding success [6]. For songbirds exhibiting loop-migration, where spring and fall routes are distinct, first-time spring migrants must navigate along a novel route to an unknown breeding site. The migration strategies of juvenile birds during this critical life history phase have been elusive. Laboratory studies and displacement experiments have provided insights into how navigational systems of juvenile birds develop over time [7], [8], but free-living juvenile songbirds have never been tracked on spring migration from start-to-finish to examine spatial and temporal patterns of migration behaviour. Differences in spring migration between adult and juvenile birds, such as the number and duration of stopovers, migratory routes, and migration timing along the route, have not been studied [9]. Recent innovations in tracking technology [10] provide the first opportunity to examine full-journey spring migration of juvenile songbirds. Now it is possible to track juvenile birds from their first wintering site in the tropics to their first temperate breeding site.

Juvenile birds nearly always arrive at breeding sites later than adults [11]–[13]. Late arrival at breeding sites can result in reduced pairing and mating opportunities [6], reduced access to high-quality breeding territories [14], and reduced opportunities to re-nest after predation [15]. Late-arriving birds may find breeding territories saturated upon arrival [16], leading to breeding dispersal or settlement in poor-quality habitat. The mechanisms for delayed juvenile arrival in spring remain elusive because most birds can only be studied at a single point on migration, without knowledge of final breeding destinations or non-breeding origin [17]. Juvenile recruitment to breeding areas has been highlighted as a major contributing factor to population dynamics in some species [18], and for one migratory songbird, most mortality occurs during migration [19].

The objective of this study was to quantify the first spring migrations of juvenile wood thrushes (*Hylocichla mustelina*) tracked by using light-level geolocators from two wintering sites (Belize and Costa Rica, n = 17) and compare them with adult migrations originating at the same sites (n = 30). Spring and fall migrations in wood thrushes occur along significantly different routes (‘loop migration’) [20]; therefore, spring migration route is truly novel for juvenile birds. Adult wood thrushes return in spring to their breeding sites, often the same territories, as in previous years. Juveniles return to their natal region, but only rarely to their hatching site. Therefore adults have experience travelling their spring migration routes, and can recognize and home-in on their exact breeding territory from previous year (s). In contrast, juveniles have only a general map of where they were hatched; the specifics of the migration route and their final destination are unknown.

Migration behaviour of adults and juveniles is predicted to differ in several important ways. There are two over-arching hypotheses that account for age-related differences in migration. The first hypothesis posits that juveniles are sub-par competitors/foragers and thus unable to attain sufficient fuel reserves for migration as early, quickly, or efficiently as adults. This hypothesis leads to the prediction that juveniles would depart later than adults from winter sites. Most wood thrushes fly across the Gulf of Mexico (800–1000 km) in spring, which is predicted to be the most energetically efficient route [21]. However, birds in poor condition are thought to avoid this risky open-water flight altogether by travelling the longer, over-land route [22]. Therefore, we predict that juveniles will be more likely to go around the Gulf than fly across owing to poor condition. Juveniles may also have lower foraging efficiency at migratory stopover sites [23], [24] and or lower flight efficiency owing to differences in wing morphology [25]–[27], and we therefore predict they will have longer and/or more frequent migratory stopovers relative to adults.

A second, non-mutually exclusive hypothesis is that juveniles have an optimal migration strategy that differs from the optimal migration strategy of adults. Game-theory models support the idea that when costs of arriving early at breeding sites are high (e.g. low chance of defending/acquiring a territory, high risk of mortality in early spring), then it could be adaptive to arrive later [16]. This hypothesis predicts that juveniles would depart consistently later on migration, regardless of condition/foraging ability, owing to an endogenous program that differs from that of adults. Birds have been shown to have endogenous programs with respect to migration preparation (lipogenesis), orientation, and even migration distance [28]. For trans-continental migrants, spring departure is presumed to be endogenously controlled [29]. It has also been shown that the experience of fall migration changes the brains of juvenile migratory birds [30], suggesting that by spring migration juveniles may be more similar to adults in their innate ability to migrate. Thus any differences in migration strategy of juveniles and adults in spring may be a result of different endogenous programs, as opposed to migratory ability. An age-related ‘adaptive migratory syndrome’ could function in a similar way to different endogenous programs of geographically separated subspecies which result in some populations exhibiting migration behaviour at different times or for different durations [31].

## Materials and Methods

### Ethics Statement

Permits from the Belize Forestry Department and the Costa Rican MINAE were obtained for all research, and the York University Animal Care Committee (York University, in Toronto, Ontario), approved bird handling and research protocols.

Wood thrushes were captured at two privately owned research stations in Central America: Belize Foundation for Research and Environmental Education (BFREE) in the Toledo District of southern Belize (16.5°N, 88.7°W), and La Selva Biological Station (operated by the Organization for Tropical Studies) in Costa Rica (10.4°N, 84.0°W). Birds were captured in arrays of 10–20 mistnets (36 mm mesh, 12×2 m), either passively, or by attracting birds to nets using wood thrush song and call audio playback. Once captured, birds were given a unique numbered metal band and a combination of colored leg bands. Since wood thrushes are sexually monomorphic during the non-breeding season, we collected a 50 µl blood sample by brachial venipuncture for genetic sexing. Blood was stored in Queen’s Lysis Buffer at 4°C, or air-dried on filter paper and stored at room temperature (24°C) until DNA extraction in the laboratory 4–6 months later. We also collected 1 tail feather and 3–4 breast feathers as back-up DNA samples. While in the hand, birds were aged as juveniles or adults following plumage characteristics described by Pyle [32]. We took digital photographs of age-specific characters of the wing and tail of each bird. Individuals with ambiguous plumage characteristics were not included in age-specific analyses.

Most captured wood thrushes received a geolocator backpack (n = 355 deployed from 2010–2013), attached by custom-fitting a Teflon ribbon leg-loop harness. Geolocators weighed 1.6 g (British Antarctic Survey/Biotrack), and including the harness, <2 g. Stalk length for the geolocator was 10 mm in 2010, and 20 mm in all other years, with an angle of ∼15°. The entire backpack (geolocator plus harness) was equivalent to approximately 4% of the body weight of the average wood thrush (mean weight: 46.50±0.19 g, n = 479). Body weight did not differ between tagged birds and non-tagged birds, nor between adults and juveniles. At our study site in Belize, mean weight of geolocator-tagged adults was 45.95±0.39 g (n = 87), and mean weight of tagged juveniles was 46.07±0.36 g (n = 115). Return rates of geolocator-tagged birds varied by year but did not differ from return rates of banded-only birds (overall 20% average return rate) [33]. A recent study documented possible effects of geolocators on migration and other behaviours of a small songbird [34]. Since we cannot directly compare the spring migration of backpack-wearing birds with controls (whose migration cannot be determined), we examined repeat captures of birds within the same winter in Belize, and compared body condition of birds that received geolocators (n = 15) and those that did not receive geolocators (n = 10), to assess if the backpack was associated with lower body condition prior to migration. We found no significant difference between geolocator and non-geolocator birds in the seasonal change in percent lean body mass (see below for methods) (Fig. S1A–C). Body condition of all individuals was higher in the wet season (Oct–Dec) and significantly lower in the dry season (Jan–Apr) (Fig. S1D). We also examined return rates of juvenile birds with and without geolocators at our study site in Belize. Return rate of banded-only juveniles was 9% (12 of 133), while return rate of geolocator-wearing juvenile birds was 8% (6 of 74). This difference was not statistically significant (Pearson’s chi-squared test, P = 1.0).

### Geolocator returns and analyses

We retrieved 62 geolocators from returning wood thrushes between 2010 and 2013. Five geolocators failed to record any data. Ten individuals were tracked in multiple years, either as a result of repeated geolocator deployments (n = 8) or because they were recaptured after carrying their geolocator for 2 years (n = 2). We preferentially used the first migrations of repeat-track birds, if available, and omitted from analysis any migration data from the additional year. Our final sample size was 47 spring migration tracks from different individuals; 17 from juveniles on their first spring migration and 30 from adults. For most birds (83% of adults and 88% of juveniles) we were able to determine the entire spring migration route. Some variables could not be measured for a given individual due to poor-quality light data, or where geolocators failed before recording the entire migration, therefore sample size varies depending upon the variable of interest from n = 15–17 juveniles and n = 24–30 adults.

To analyze the geolocator light data, we used a threshold approach, calibrated by live ground-truthing at breeding and wintering sites to determine sun elevations [35]. For spring migration, we used a breeding-grounds based sun elevation angle to calibrate locations, following McKinnon et al. [35]. One person (E. A. M.) analyzed spring migrations of all birds independently, and without knowledge of age or sex of the bird, to avoid any observer bias in interpretation of geolocator data. We used ordinal date for all analyses, such that January 1 = 1. We relied primarily on longitude to determine movements of wood thrushes, since it is more reliable and accurate than latitude [36]. Latitudes cannot be estimated for approximately two weeks pre- and post-vernal equinox. Longitudes that shifted by >2° were considered migration movements, except for crossing the Gulf of Mexico, which was evident by a large jump in latitude (10–15°), often with little movement in longitude. Gulf crossing occurred >2 weeks after the vernal equinox (i.e. after 2 April) for all birds.

Timing of migration was determined for each individual at three points: last noon at winter site, timing of crossing into the U.S. (date of first noon across 23.5°N) and first noon at breeding site. Stopovers were defined for each individual as two or more consecutive noon fixes that differed by less than 2° in longitude. Two consecutive noons in the same location were considered 1 stopover night (i.e. one night with no migration; wood thrushes are nocturnal migrants). Total stopover nights were summed for each bird, and the location of each stopover was divided into two categories – ‘tropical’ (<23.5°N) or ‘U. S.’ (>23.5°N). We looked specifically at the duration of the last stopover in the tropics and the first stopover in the U. S. to assess if juvenile birds need to stopover for longer before or after the open-water 800–1000 km Gulf of Mexico crossing. Since many wood thrushes use the Yucatan peninsula to cross into the temperate breeding region, we used the longitude of entry into the U. S. at the north coast of the Gulf of Mexico to quantify migration route. At this point, approximately 30°N, wood thrushes have a relatively broad land-base (80–95°W) across which they could enter the U. S. and move to their breeding site. We calculated the average latitude and longitude for June and July for each individual to determine its breeding region. To determine migration speed for each bird, we measured overall spring migration distance by connecting its winter site to each stop, ending at its breeding site, by using straight lines. We then divided distance by the total duration of migration in days to get overall migration speed (km/d). To determine speed on flight nights only, we divided migration distance by duration minus the number of stop nights.

### Condition and morphology analyses

To determine if age differences in body condition prior to spring migration could drive differences in migration behavior, we examined the condition of adult and juveniles birds captured during the dry season (Jan–Apr) at our study site in Belize, Central America in 2011 and 2013. For each bird, we recorded mass to the nearest 0.1 g using a Pesola spring scale, and measured the right metatarsus bone length (hereafter, ‘tarsus’) to the nearest 0.1 mm using Vernier calipers. One researcher (E. A. M.) conducted all field measurements for consistency. We used tarsus measurements for condition analyses because they were the most significant predictor of fat-free body weight for our dataset, when compared to other linear measurements (bill length, wing length, tail length, or the first principal components calculated from all measurements) [37]. We also recorded fat score (scale of 0–7, based on MoSI protocol [38]), pectoral muscle score (0–2, based on [39]). We regressed weight of birds with fat score of zero (n = 45) against tarsus length to derive the following equation: lean body mass = 12.34+1.03*tarsus length. We then used this equation to calculate a ‘predicted’ lean body mass (PLBM) for each individual based on its tarsus measurement. The difference between actual mass and PLBM was calculated, and converted to a percent relative to PLBM for use as a condition index (% PLBM), following methods by Bayly et al. [40]. We used a general linear model to examine age effects on % PLBM, and included age, sex, and date of capture as factors. We also examined pectoral muscle scores and fat scores by age, including sex and capture date as covariates.

We measured unflattened right wing chord to the nearest mm for adults (n = 145) and juvenile (n = 191) birds from our study site in Belize. Wing length differed significantly by sex; therefore we tested for differences by age separately for males and females by using t-tests.

### Statistical analyses

We used general linear models in the program R [41] to determine if age was a significant predictor of spring migration timing (at three points: departure, crossing into the U. S. and arrival at breeding sites) and spring migration route (longitude at entry into the U.S., and migration distance). All full models for migration timing and route included the following independent factors: age, sex, and breeding location (latitude and longitude), as well as an interaction between age and sex. We included breeding destination and sex as factors in the models since birds migrating farther (i.e. to the northern breeding range), and males (which arrive first at breeding sites) are likely to show different migration behaviour than birds migrating shorter distances, and females. We also included an interaction term for age and sex to account for potential differences in migration strategy by sex within-age class. We used the function “step” in R to drop terms and determine the best-fit model using AIC values.

For stopover variables we used a different approach to summarize age-related differences, since initial examination of the data revealed no sex or breeding destination effects. We used age as the response variable in a binomial linear regression and used the following stopover variables as independent factors: total stopover nights, stop nights in the U. S., stop nights in the Tropics, mean stopover duration, number of stops, and duration of stopover before Gulf crossing and after gulf crossing. This analysis allowed us to assess which stopover variable was the most different between adults and juveniles (i.e. significant predictor of age). We also compared stopover variables directly between adults and juveniles using t-tests in order to show the mean differences for each variable. We examined variance in longitude entering the U. S. between adults and juveniles using Fisher’s *F* test, and we compared whether or not birds crossed the Gulf of Mexico overwater or took an over-land route by using Fisher’s Exact test to determine if juveniles were significantly more likely to go around the gulf than adults. We report means ± standard error unless otherwise indicated.

## Results

### Migration timing

**Figure 1:**
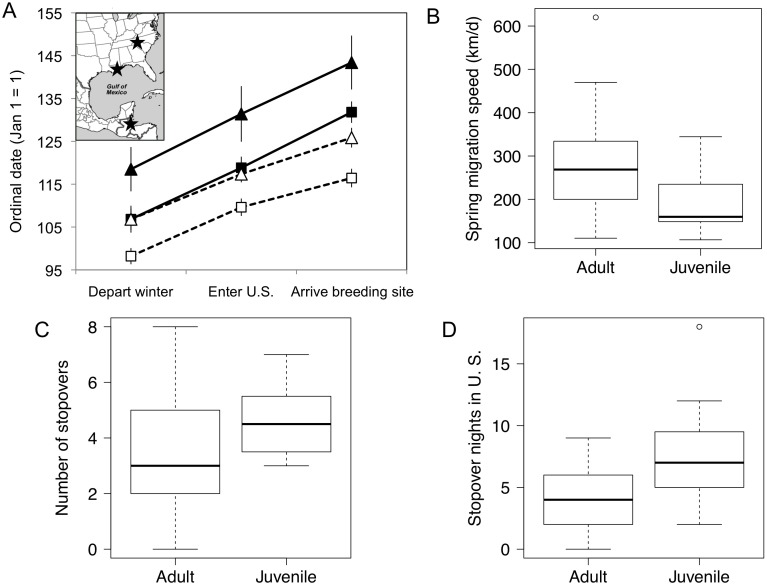
Juvenile wood thrushes exhibit a significantly different migration strategy in spring than adults: A) Juveniles (solid symbols) were later than adults (hollow symbols) at winter departure, entering the U. S. along the northern Gulf of Mexico coast, and when arriving at breeding sites. Triangles represent average for females and squares for males. Vertical bars indicate standard error. Inset map shows an example of locations where timing was measured for an individual wood thrush tracked from Belize. B) Spring migration speed (total distance/duration) was slower for juvenile wood thrushes. C) Juveniles had more stopovers during spring migration relative to adults, and D) had more stopover nights in the U. S. than adults. Note that 1 stopover night = 2 consecutive noons in the same location. Boxplots extend to 25^th^ and 75^th^ quartiles with dark lines showing the median value and circles indicating outliers. Sample size is n = 17 for juveniles, and n = 30 for adults, except for winter departure (n = 16, 26) migration speed (n = 15, 25), and stopovers in the U. S. (n = 16, 29).

Juveniles departed significantly later from overwintering sites than adults (model estimate for juveniles = 7.9±2.9 d, P = 0.009) (Fig. 1A), controlling for significant sex and breeding destination effects (Table 1). This timing difference was also evident at the U. S. Gulf of Mexico coast, where juveniles also arrived later relative to adults (estimate for juveniles = 9.02±2.7 d, P = 0.001) (Fig. 1A). Age had the most pronounced effect on timing of arrival at breeding sites (estimate for juveniles = 13.97±2.7 d, P<0.001) (Fig. 1A). There was no significant interaction between age and sex for any timing variable.

**Table 1:** Top general linear models that explain variation in spring migration behaviour of juvenile and adult wood thrushes.

Dependent variable	Factors retained in top model	Estimate±standard error	t	P
Spring departure date	Age (J)	7.93±2.91	2.73	0.009
	Sex (M)	−10.88±2.89	−3.76	<0.001
	Breeding latitude	0.74±0.38	1.96	0.057
Spring enter U.S.	Age (J)	9.02±2.66	3.39	0.001
	Sex (M)	−10.57±2.53	−4.17	<0.001
	Breeding latitude	1.02±0.34	2.98	0.005
Breeding arrival date	Age (J)	13.97±2.66	5.25	<0.001
	Sex (M)	−11.58±2.53	−4.57	<0.001
	Breeding latitude	1.12±0.34	3.03	0.002
Spring migration duration	Age (J)	7.73±2.74	2.81	0.008
Longitude entering N.A.	No significant factors	-	-	-
Spring migration distance	Breeding latitude	79.78±26.81	2.98	0.002
	Breeding longitude	−107.49±25.81	−4.16	<0.001
Spring migration speed(distance/migration duration)	Age (J)	−80.70±33.99	−2.37	0.023

Letters in brackets indicate the base category for that estimate, i.e. J = juvenile, M = male. Full models for all variables included age, sex, breeding latitude and breeding longitude. We also included an interaction term for age and sex, although it was not significant in any model.

### Spring migration duration and speed

Age was the only significant factor retained in the top model for spring migration duration (estimate 7.73±2.74, t = 2.81, P = 0.008) (Table 1), indicating that the effect of age on spring migration duration is larger than effects of location of the final destination, or sex of the bird. Juveniles spent, on average, about 8 d longer on spring migration than adults (mean 24.5±2.5 d for juveniles versus 16.8±1.5 d for adults). Overall migration speed (calculated as the total distance covered divided by the duration in days) was significantly slower for juveniles (191±18 km/d) than adults (272±24 km/d) (t = 2.60, df = 35.58, P = 0.014) (Fig. 1B). Migration speed on flight nights only was not significantly different by age (juveniles: 541±39 km/d, adults 592±35 km/d; t = 0.98, df = 33.04, P = 0.33).

### Migratory stopovers

**Figure 2:**
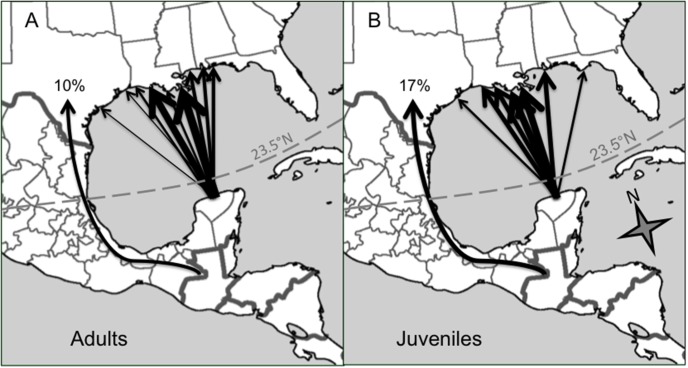
Migration routes at the Gulf of Mexico are not significantly different for A) adult (n = 30) and B) juvenile (n = 17) wood thrushes. Lines point to the longitude on the northern Gulf of Mexico coast where birds made landfall. Thickness of a line is proportional to the percent of birds within age-class using that route. Birds are all shown crossing the Gulf from a single point on the Yucatan peninsula of Mexico for simplicity; there were slight variations in takeoff locations that are not shown here. The arrow around the Gulf indicates the proportion of birds that did not fly directly across but instead used an overland route (n = 3 of 17 juveniles, n = 3 of 30 adults).

The only significant factor retained in our binomial linear regression with age as the dependent variable was total number of nights stopped in the U. S. (estimate for juveniles: 0.94±0.35, z = 2.69, P = 0.007). Comparing stopover variables between adults and juveniles using *t*-tests revealed that the frequency of stopovers was significantly different by age (t = −2.46, df = 38.51, P = 0.018): juveniles stopped more times (4.6±0.3) than adults (3.4±0.3) (Fig. 1C). In contrast, mean stopover duration was not significantly different between adults and juveniles (mean for juveniles: 3.4±0.3 d, mean for adults 3.2±0.3 d; t = −0.58, df = 35.22, P = 0.56). Juvenile birds had more stop nights over the entire migratory journey (juveniles: 16.2±2.1 d, adults: 10.2±1.1 d; t = −2.44, df = 24.51, P = 0.02), and more specifically, more stopover nights in the U. S. (juveniles: 7.4±1.0 d, adults: 3.9±0.5 d; t = −3.09, df = 21.989, P = 0.005) (Fig. 2D). There was no significant difference between adults and juveniles in the number of nights spent at stopovers in the Tropics (juveniles: 8.8±2.0 d, adults: 6.6±1.1 d) (t = −0.90, df = 24.61, P = 0.38). We found no age-related differences in the duration of the last stopover before the Gulf of Mexico crossing (t = −0.08, df = 33.97, P = 0.93), or in the duration of the first stopover on the U.S. side (t = −0.98, df = 22.75, P = 0.34).

### Migration route and destination

Age was not a significant factor explaining variation in longitude of entering the U. S. (Table 1). Since there were no significant effects of breeding destination or sex on longitude entering U. S., we directly compared the longitude of adults versus juveniles at the point of entry to U. S. by using a t-test, and did not detect a significant difference by age (juveniles: 91.7±0.7°W, adults: 90.6±0.5°W; t = −1.20, df = 30.33, P = 0.24) (Fig. 2). Variance in longitude entering the U. S. was also not significantly different by age (F = 0.80, df = 29/16, p = 0.58). Migratory distance, controlling for breeding destination, was also not significantly different by age, as would be expected if juveniles took longer routes to get to the same place (Table 1). A slightly higher proportion of juveniles avoided crossing the Gulf of Mexico overwater (3 of 17) compared to adults (3 of 30), although this was not significant (Fisher’s Exact test, P = 0.65) (Fig. 2).

### Condition and morphological differences prior to spring migration

**Figure 3:**
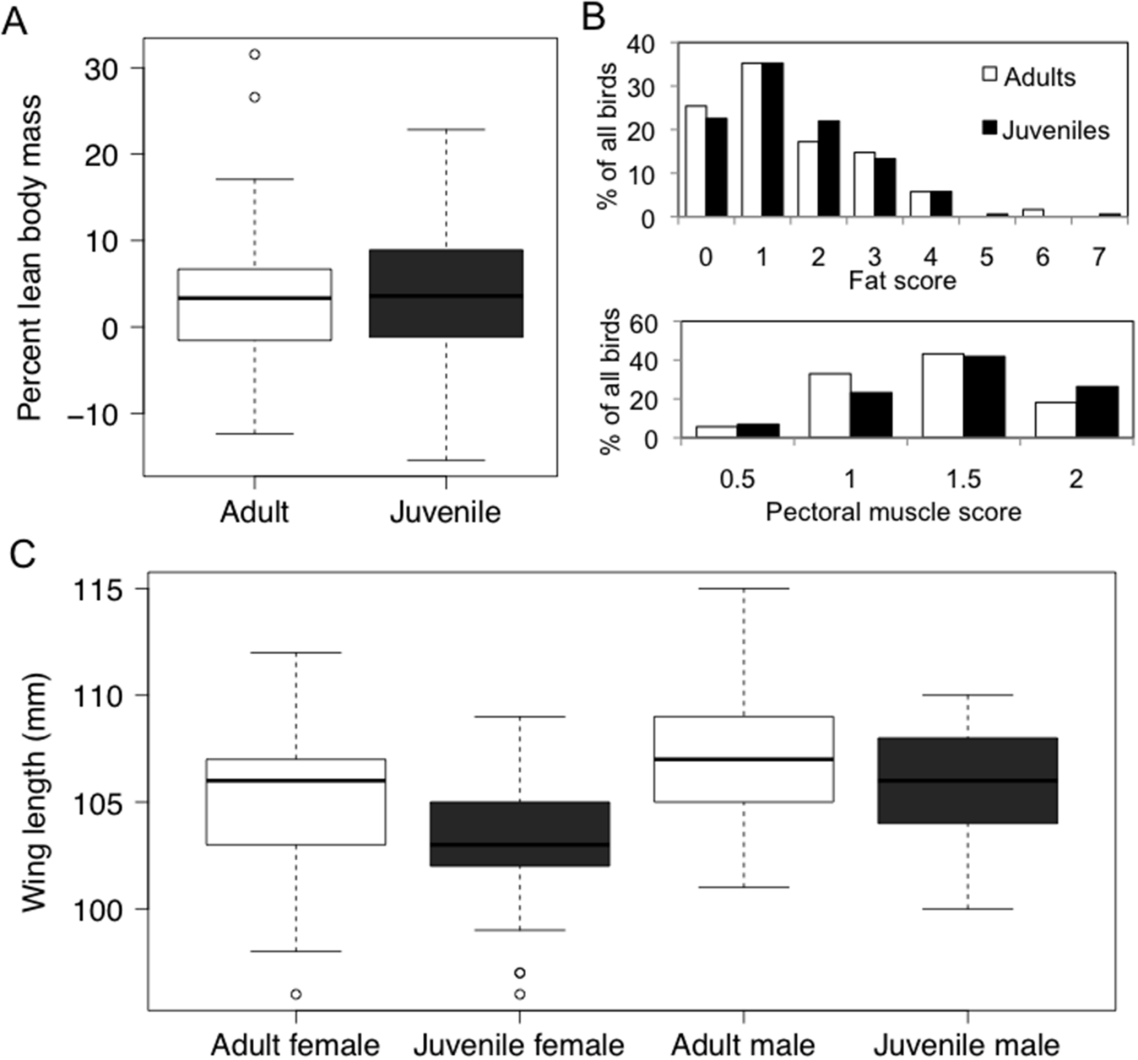
Body condition of adult (n = 78) and juvenile (n = 107) wood thrushes captured in Belize in late winter was not significantly different, but wing length was significantly shorter for juveniles, within sexes. A) Percent lean body mass, and B) fat (n = 122 adults, n = 173 juveniles) and pectoral muscle scores (n = 88 adults, n = 129 juveniles) were not different by age-class. C) Wing length was smallest for juvenile females (n = 92), followed by adult females (n = 73), juvenile males (n = 99), and adult males (n = 72). Boxplots show 25^th^ to 75^th^ quartiles with open circles indicating outliers.

Age was not retained in the top model for percent lean body mass (P = 0.23), nor was there a significant interaction between age and date of capture (P = 0.22) in Belize. Mean %PLBM for adults was 3.45±0.87% and for juveniles 4.04±0.73% (Fig. 3A). We also did not find age-related differences in two other measures of body condition, pectoral muscle scores (F = 2.65, P = 0.106) and fat scores (F = 1.581, P = 0.210) (Fig. 3B). Juvenile wood thrushes had significantly shorter wings than adults, within sex-class (females: t = 4.95, df = 135, P<0.001; males: t = 3.78, df = 132.39, P<0.001) (Fig. 3C).

## Discussion

For the first time, we tracked juvenile songbirds from start-to-finish on their inaugural spring migration and found significant differences in migration strategy between juveniles and adults tracked from the same wintering sites. After accounting for the effects of sex and breeding destination, juveniles were a week later than adults in departing from wintering sites and two weeks later in arriving at breeding sites (Fig. 1A). The slower migration speed of juveniles overall (Fig. 1B) was the result of juveniles stopping for more nights than adults (Fig. 1C, D), which resulted in a ∼50% longer spring migration duration for juveniles. Juveniles did not take significantly longer routes, or significantly different migration paths at the Gulf of Mexico, nor were they more likely to go around a major ecological barrier (Fig. 2).

Differences in spring migration departure date from the tropics suggest either that juveniles are unable to optimally respond to photoperiod cues in spring, or that they respond to later or different migration initiation cues, relative to adults. Photoperiod may be only one of several synchronizers that entrain endogenous circadian rhythms controlling migratory activities [42]. The availability of food has a large impact on body condition and timing of spring migration of an insectivorous migrant, the American redstart (*Setophaga ruticilla*) [43]. Food-rich habitat (mangrove forest) is monopolized by territorial, dominant adult males [44] and adults precede young birds on spring migration [17], with subsequent carry over effects on breeding success [14]. In wood thrushes, there is no evidence for age-related dominance patterns during the non-breeding season [45]. Nevertheless, juveniles could be less efficient at foraging than adults, resulting in delayed departures for younger birds if they are slower to achieve appropriate levels of fat and muscle for migration [11]. We did not find evidence of age-related differences in percent lean body mass, fat scores, or pectoral muscle scores, during late winter (Fig. 3). Our results suggest that age-related differences in condition at winter sites are not driving the overall age patterns in migration timing observed for wood thrushes. However, within-age-class variation in our study was large (range of ∼2 weeks in departure dates within age and sex classes), and future studies should examine if this variation could be explained by environmental factors.

Multi-year migration tracking of adult wood thrushes showed that departure date from wintering sites, and arrival date at the breeding site, was repeatable for an individual from one year to the next [46]. We found that first-time spring migrants differ substantially in timing from experienced migrants. This suggests that a shift from “late” to “early” migration strategy occurs at the individual level between the first and second spring migration. An age-dependent shift in migratory orientation and route has been documented in raptors and shorebirds during fall migration [47], [48]. If this difference is not related to body condition of the birds, it is possible that an endogenous timing mechanism is the primary controller of spring migration departure in juveniles. Migration of juveniles in fall is under strong endogenous control [28], [42]. Laboratory studies of migration behavior (‘*zugunruhe*’) under a constant photoperiod and resource access, similar to recent experiments with Northern wheatears (*Oenanthe oenanthe*) that showed activation of migratory behaviour in males earlier than females [49], would elucidate whether juvenile birds have an innate, later migration program relative to adults.

Arriving later at breeding sites could have evolved as an adaptive strategy for juvenile birds [16]. If there are high costs of migrating early, such as mortality from inclement weather, or aggressive encounters with older territorial individuals [11], [50], [51], selection may favour a later endogenous program for juveniles to avoid such early-migration risks. Juveniles in general are subdominant to adults in many species, thus they would probably have a low chance at holding a breeding territory when faced with an experienced adult competitor. In rose-breasted grosbeaks (*Pheucticus ludovicianus*), juveniles with more adult-like plumage preceded more typical juvenile-plumage birds on spring migration [52], suggesting that social dominance (signalled by plumage coloration) affected arrival patterns. One scenario supported by both evolutionary and game theory models is a two-wave spring arrival pattern [16], [53], with different costs and benefits for early- versus late-arriving birds. This two-wave pattern could correlate with age, if adult birds arrive in the first peak, and juvenile birds arrive during a second wave. Juveniles could therefore avoid the increased risk of arriving early and spend more time *en route* physiologically preparing for their first breeding season [54].

More stopovers in the U. S. for juveniles (Fig. 1C, D) could be explained by a need for more frequent refuelling, if foraging efficiency is lower for juvenile birds [23]. However, some studies at stopover sites have found no evidence for age-related differences in condition [55] or refuelling rates [56]. If juveniles were less efficient at refuelling relative to adults, we would expect to see longer stopover duration or more frequent stopovers over the entire migratory route, as well as longer stops before or after the ∼1000-km open-water crossing of the Gulf of Mexico. Our data indicate that juveniles do not stay longer at migration stopover sites than adults (mean duration of stopovers was similar), nor do they spend more time preparing for or recovering from an open-water crossing of ∼1000 km. It is possible that juveniles stop more frequently in the U. S. because of an adaptive strategy to conserve or acquire resources for breeding, in contrast to a final ‘sprint’ migration in which birds exhaust resources in a final push to arrive early [54]. The accuracy of geolocators is currently insufficient for mapping the precise location of stopover sites. If exact stopover locations were known, remote sensing could be used to assess habitat quality of stopover sites for juveniles versus adults. Recently-developed miniature archival GPS loggers, which have a resolution of <1 km and can sample up to 50 locations throughout the annual cycle, could be applied to further explore differences (or similarities) between adult and juvenile spring migration stopover behaviour.

Despite the inherent differences in experience between adults and juveniles, we did not detect any age-related differences in spring migration route. Tracking technology for small birds is currently limited in its ability to record fine-scale details of migration route. When higher resolution technology becomes available, age-related differences may become apparent. It is also possible that in wood thrushes, there are no significant differences in migratory routes by age in spring. Migratory birds can display long-distance homing abilities during their first spring migration; displaced juvenile European starlings (*Sturnus vulgaris*) managed to find their way to their natal site even after wintering in the ‘wrong’ place [8]. In order to navigate ‘home’ in spring, juveniles birds require a period of familiarization with their natal site before departing on fall migration, which may occur as short nocturnal flights prior to fall migration departure [57]. Thus, in spring, juveniles can use both their innate magnetic compass and navigational homing abilities to reach their destination [58]. We expected juvenile birds to take longer routes, or less risky routes (i.e. around the Gulf of Mexico, instead of across), but this was not the case. Many songbirds take relatively direct routes in spring to minimize time spent on migration and advance spring arrival dates [59]. It is possible that juveniles and adults have convergent spring migration routes because of prevalent wind and weather patterns at the Gulf in spring and an innate migratory program for when and where to migrate.

Juveniles had significantly shorter wings in our study population. Wing length is related to flight efficiency [25], and it has been hypothesized that juvenile birds trade-off flight speed for maneuverability to avoid predation [26]. We broadly estimated migration speed (or ‘rate’, [20]) as the total migration distance divided by overall migration duration, following other migration studies (e.g. [60]). Juveniles were significantly slower overall than adults (Fig. 1B); however, this appeared to be due to more frequent stopovers taken by juveniles and not slower flight speed on a given night. Thus juveniles are capable of covering the same distance as adults during each migration night. Juveniles might stop more if each flight requires more days of refueling owing to greater energetic costs of flight. Shorter wings of juveniles could lead to less efficient flight. It is important to note that we measured only wing length and not wing shape; relative pointedness is an important component of wing efficiency [25]. It is also possible that adults are better able to select appropriate tail winds and therefore decrease energy expenditure while flying. Finally, it could be that juvenile birds have an innate program to stop more frequently as they approach their breeding region, in order acquire more resources for reproduction [54]. Radio-tracking studies following birds during nightly flights (e.g. [61]) could compare distance, speed, and fuel-use of juveniles versus adults directly to determine exactly how migration differs by age at a scale of a single migratory flight.

Wood thrush populations have declined on average by ∼2%/year over the past 50 years [62]. There is evidence that most mortality in songbirds occurs on migration [19], and a recent tracking study of raptors showed that mortality during migration (both spring and fall) was six times higher than during stationary periods of the annual cycle [63]. This period could be especially limiting for juvenile birds, which we show spend ∼50% more time on spring migration. It is also possible that juvenile birds migrate using a mortality-minimizing strategy, instead of the typical adult time-minimization strategy. Regardless, understanding demographic patterns in migration is a critical step for full-life cycle modelling of migratory species to determine where conservation funds should be prioritized to mitigate further population declines.

## Supporting Information

Figure S1 **Four plots examining effects of geolocators on body condition of birds, as described in the Materials and Methods section of the manuscript.** Within-winter recaptures of birds wearing geolocators (‘Geo add’) (n = 15) and those which did not receive a geolocator (‘No geo’) (n = 10) did not show differences in percent lean body mass, and in general, all birds declined in body condition as the winter progressed from wet season (Oct–Dec) to dry season (Jan–Apr). A) Birds that were relatively heavy on first capture tended to lose more mass over time, and the same pattern was shown in both ‘Geo add’ and ‘No geo’ groups. B) Birds recaptured after more days tended to decrease slightly more in percent lean body mass, with no effect of geolocators. C) Individual birds showed no consistent pattern in changes in percent lean body mass from first to second capture. Symbols indicate: ‘Geo add’ (filled), ‘No geo’ (hollow), males (squares), females (triangles), juveniles (dashed lines), and adults (solid lines). D) Average percent lean body mass of all birds was significantly higher in the wet season (n = 66) relative to the dry season (n = 88) (vertical lines show standard error).
(TIFF)
